# Supplementary figures and images for: Investigating mechanisms underlying genetic resistance to Salmon Rickettsial Syndrome in Atlantic salmon using RNA sequencing
Source: BMC Genomics. 2021 Mar 6;22:156. doi: 10.1186/s12864-021-07443-2 (PMC7936450; doi:10.1186/s12864-021-07443-2)

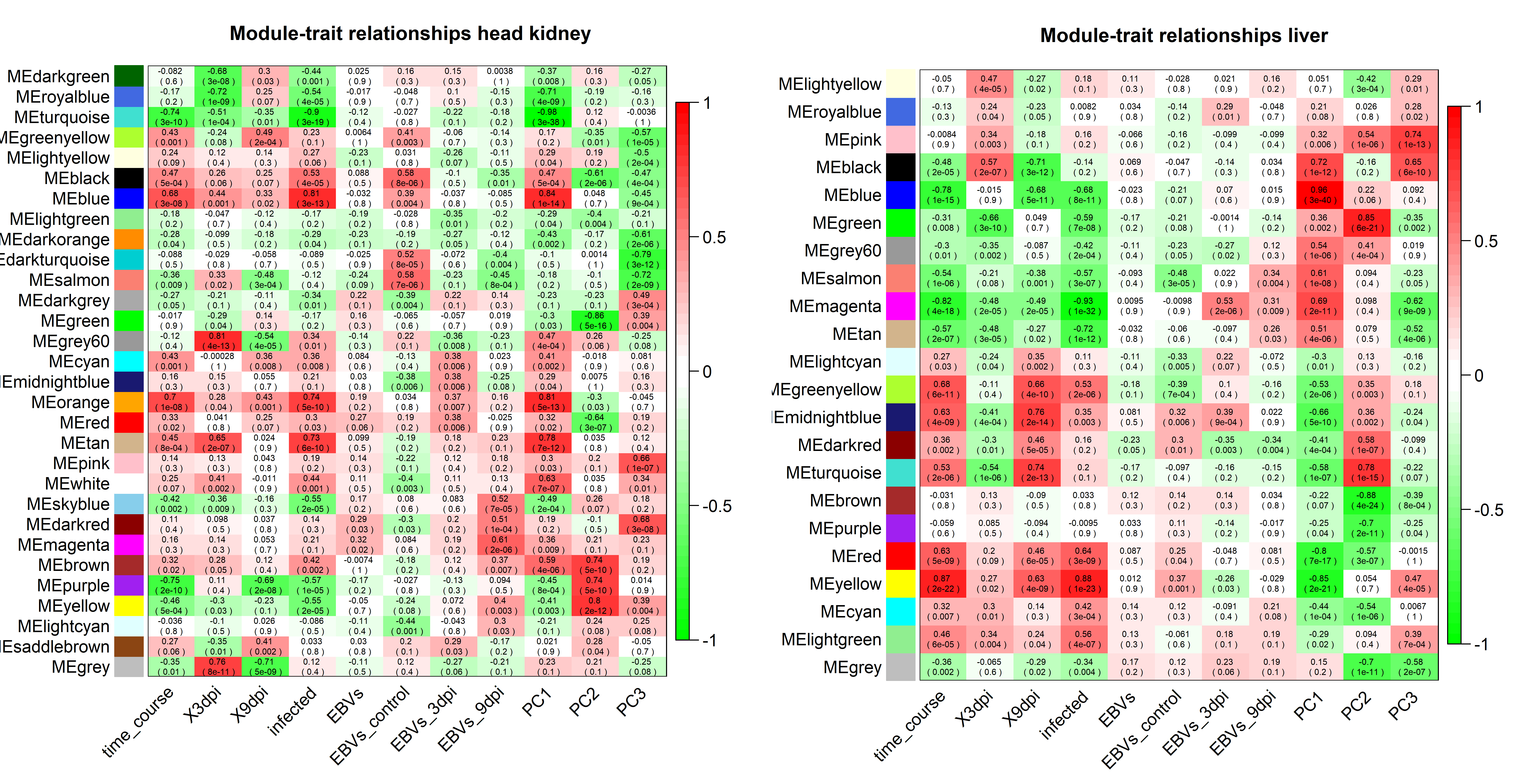

Supplement: Supplementary file 6 — Additional file 6: Supplementary Figure 1. Correlation between different phenotypes and the gene expression pattern of the WGCNA networks in head kidney and liver [file 12864_2021_7443_MOESM6_ESM.png]
